# Attractiveness and determinants of different tobacco products among Chinese smokers and non-smokers: a web-based cross-sectional study

**DOI:** 10.3389/fpubh.2026.1763854

**Published:** 2026-02-24

**Authors:** Ting Fei, Dawei Yan, Xiaomin Liu, Yuyan Li, Yihan Gao, Xiaonan Li, Guangchao Liu, Bingxue Wu, Yishi Jiang, Yan Zhang, Yan Che, Saijing Zheng

**Affiliations:** 1 Shanghai New Tobacco Product Research Institute Co., Ltd., Shanghai, China; 2 NHC Key Lab of Reproduction Regulation, Shanghai Engineering Research Center of Reproductive Health Drug and Devices, Shanghai Institute for Biomedical and Pharmaceutical Technologies, Shanghai, China

**Keywords:** appeal score, China, combustible cigarettes, non-smokers and smokers, novel tobacco products

## Abstract

**Background:**

Tobacco use remains highly prevalent in China, with the emergence of a growing range of tobacco and nicotine products that vary in design and social presentation, raising questions about how these product characteristics are perceived by consumers. This study assessed the attractiveness of five tobacco products among the general Chinese public, including both non-smokers and smokers, and explored the factors influencing their perceived appeal.

**Methods:**

Using a self-designed attractiveness scale, an online survey was administered via smartphone to adults aged 18 years or older across six geographically representative cities in China (Beijing, Shanghai, Guangzhou, Shenzhen, Chengdu, and Zhengzhou). The survey assessed the attractiveness of five tobacco products, including combustible cigarette (CC), e-cigarette (EC), heated tobacco product (HTP), oral nicotine product (ONP) and tobacco-free oral nicotine pouch (TFONP). Attractiveness ratings for different tobacco products were calculated, and mixed linear models were employed to explore associated influencing factors.

**Results:**

A total of 15,601 valid questionnaires were collected, including 9,700 non-smokers and 5,901 smokers. CC had the highest familiarity score (3.97 ± 1.04) and were perceived as having the highest health risks (4.38 ± 0.76) and the highest addictiveness (4.22 ± 0.87). All alternative tobacco products received significantly lower ratings on these measures compared with CC (all *p* < 0.05). Total attractiveness scores were similar for CC and e-cigarettes (both 3.04) but lower for HTP/ONP/TFONP (range: 2.59–2.63; all *p* < 0.05). In the multivariable Model 1, which did not include “perceived health risks,” smoking status showed no significant association with attractiveness (*β* = 0.001, *p* = 0.896). After incorporating this variable in Model 2, the association became modestly negative and statistically significant (*β* = −0.06, *p* < 0.001). Relative to CC, e-cigarettes were associated with higher attractiveness ratings (*β* = 0.14), while HTP, NOP, and TFONP were associated with lower attractiveness ratings (*β* = −0.09 to −0.14; all *p* < 0.001). Use history exhibited the strongest association with attractiveness (β = 0.56). In addition, higher “familiarity with tobacco products,” “perceived social support” and “perceived addictiveness” were positively associated with product appeal, whereas greater “perceived severity of withdrawal” and “perceived health risks” were negatively associated with appeal. Attractiveness ratings also varied somewhat across cities, and gender, age, and occupation had certain influences on the ratings.

**Conclusion:**

This study identified distinct patterns of perceived appeal across five tobacco/nicotine product types among Chinese adults. E-cigarettes received the highest appeal ratings among non-smokers, whereas combustible cigarettes remained the most appealing among CC users. Prior product use history showed the strongest association with appeal. Perceived health risks were inversely associated with appeal and appeared to partly account for the smoking–appeal association, as the association between smoking status and attractiveness ratings changed after adjustment for perceived health risks. In contrast, greater familiarity and higher perceived social support were positively associated with appeal.

## 1 Introduction

Recent decades have witnessed a continued decline in overall tobacco use worldwide, yet the rapid proliferation of novel nicotine and tobacco products, such as electronic cigarettes (ENDS), heated tobacco products (HTPs), and other emerging products (1). China is the world’s largest tobacco-consuming nation (2). In 2024, China’s smoking prevalence among adults aged ≥15 years was 23.2% (current) and 20.3% (daily), with current smokers averaging 15.8 cigarettes/day and daily smokers initiating daily smoking at 22.4 years, and slim cigarette use accounted for 32.9% of current smokers (3). Although only 2% of women in China now smoke, half of all adult men smoke cigarettes (4). While combustible cigarette smoking continues to account for the majority of tobacco use, Chinese consumers are also exposed to a growing diversity of nicotine and tobacco products and related marketing narratives (5, 6). In recent years, China’s regulatory landscape for novel tobacco products has evolved substantially. The mandatory national e-cigarette standard took effect in October 2022 (7), alongside strengthened licensing, transactional regulations, and a market restriction to tobacco-only flavors, significantly reshaping product availability and marketing (8). Within this context, “product appeal”—encompassing sensory experience, perceived benefits or risk, marketing cues, and psychosocial meanings—has emerged as a key construct for understanding experimentation and continued use across product categories (9).

Studies suggest that e-cigarettes may be perceived as appealing due to novelty flavors, convenience, and ease of use, which may contribute to experimentation (10), and HTPs highlight appeal dimensions such as innovation, substitutability for cigarettes, and youth appeal, although use intention may remain low in some samples (11). In China, university students—particularly males—appear more likely to endorse social or psychological advantages of alternative nicotine/tobacco products (12). Overall, appeal is product-specific and context-dependent, underscoring the need to compare perceived attractiveness across multiple products within China. To move from description to explanation, determinants of appeal can be organized into three interrelated domains: (i) Product Appeal and Perceived Vulnerability, capturing how attractiveness interacts with perceived susceptibility to shape awareness and behavioral vulnerability (13–16); (ii) Psychosocial Appeal, including motivations related to social belonging, group identity, and psychological needs (17, 18); and (iii) External and Contextual Influences, such as the policy/regulatory environment (7, 8) and marketing-related factors (17, 19). These domains are linked through individuals’ risk appraisal and health beliefs: higher appeal may attenuate perceived threat or weaken protective responses. Rooted in Protection Motivation Theory (PMT) and the Health Belief Model (HBM), this pathway encompasses perceived severity, susceptibility, response efficacy, and self-efficacy (20, 21).

Evidence regarding perceived attractiveness of different tobacco/nicotine products among the Chinese public, and the factors shaping such attractiveness, is limited (12, 15). To address this gap, we adopt an integrated perspective that combines multi-dimensional appeal (product-related, marketing-related, and psychosocial appeal) with risk appraisal (22). Specifically, PMT and HBM are used to structure the risk appraisal pathway, capturing how perceived severity, susceptibility, and efficacy beliefs relate to perceptions of tobacco-related threats (20, 21). Appeal is conceptualized as a parallel pathway that may directly influence interest and trial and may also interact with risk appraisal (6, 9). Higher appeal is expected to be associated with lower perceived threat (e.g., reduced perceived severity/susceptibility) or to attenuate the translation of threat appraisal into avoidance intentions (20, 21). Accordingly, this study aims to (i) compare perceived attractiveness across five tobacco/nicotine product types between smokers and non-smokers and (ii) identify key correlates of attractiveness.

## 2 Methods

### 2.1 Types of tobacco products

Viewing of tobacco products use in China, this study examined five types of tobacco products: combustible cigarettes (CCs), e-cigarettes (ECs), heated tobacco products (HTPs), oral nicotine products (ONPs) and tobacco-free oral nicotine pouches (TFONPs).

### 2.2 Sample size

The sample size was determined to ensure adequate statistical power for within-respondent comparisons of appeal across five product types (paired design) and to support multivariable analyses of correlates. Based on a 5-point Likert scale, and in the absence of prior standardized measures, we assumed a minimum detectable difference of 0.2 points, a standard deviation of 1.0, *α* = 0.05, and 90% power. Using PASS 2021 (paired *t*-test), the required sample was 259 participants. Additionally, following the rule of thumb of 10–20 observations per item (maximum 10 items) (23), a minimum of 200 participants was indicated.

To ensure coverage of key subgroups, quota targets were set by region (six cities across China), smoking status, and gender. Within each city, 2,500 respondents were targeted, with gender ratios prespecified to account for China’s smoking-prevalence disparity (male smokers: male non-smokers = 1:1; female smokers: female non-smokers = 1:3). This design facilitated comparisons across subgroups, but the sample is not a probability-based representation of the national population. The sampling strategy enabled efficient nationwide recruitment and within-respondent comparisons within a single survey session. As participation was voluntary and smartphone-based, the sample should not be interpreted as a probability sample. Therefore, sample-size calculations justify power for detecting within-respondent differences and modeling appeal correlates, not for population representativeness.

### 2.3 Study participants

This study used an online, non-probability sample collected via an electronic data collection (EDC) system (Wenjuanxing platform, a widely used online survey platform in China). Participants were adults aged 18 years or older. Inclusion criteria were: (1) aged ≥18 years; (2) able to use a smartphone and complete surveys via mobile applications; (3) able to read and complete the questionnaire independently; and (4) provision of informed consent. Individuals were excluded if they were unable to operate a smartphone or unable to read or complete the questionnaire independently.

Given the requirement for smartphone access and independent completion of an online questionnaire, the final sample is expected to over-represent younger and more highly educated respondents relative to the general adult population. To transparently account for this limitation, we report detailed participant demographics and include age and education as covariates in all multivariable models. Findings are therefore interpreted primarily as evidence on comparative appeal patterns and their correlates among smartphone-using adults, rather than as estimates of population prevalence.

### 2.4 Study contents

#### 2.4.1 Demographic characteristics

Five variables, including gender, age, education, occupation, and monthly household income per month were investigated.

#### 2.4.2 Tobacco product use history and risk perception

Six variables were included in this study, according to some literature (24–26). (1) Tobacco product use history (“whether tobacco product have ever been used,” answer yes or no); (2) Familiarity (“Before this survey, how familiar were you with [cigarettes/ECs/HTPs/ TFONP / TFONP]?”); (3) Health risk perception (“To what extent do you believe using *[product]* is risk for your own health?”); (4) Addictiveness perception (“People are likely to become addicted to *[product]*”); (5) Perceived quitting difficulty (“It is easy for people to quit using *[product]*”); and (6) Social facilitation perception (“Using *[product]* is helpful for social interaction”).

The 5-point Likert scale (27) was used as follows: for *Familiarity*: 1 = “Extremely unfamiliar” to 5 = “Extremely familiar”; for *Health risk perception*: 1 = “None” to 5 = “Extremely high”; and for the remaining three dimensions (*Addictiveness perception*, *Perceived quitting difficulty*, and *Social facilitation perception*): 1 = “Strongly disagree” to 5 = “Strongly agree.”

#### 2.4.3 Attractiveness survey questionnaire design

Building on previous studies (10, 14, 16) and the 5-point Likert scale (27), the framework incorporates four focal dimensions: health risk decision-making (10, 16) (including personal health risks and risks to others), sociocultural factors (28, 29) (such as social factors and “cool culture”), product engineering attributes (10, 14, 16, 30) (e.g., method of use, appearance, design and convenience) and neurosensory experience (10, 31) (e.g., product feel & sensation and physiological satisfaction). Physiological satisfaction was only assessed among respondents with a history of tobacco product use (see Supplementary Table S1A for items and Supplementary Table S1B for sample questions). The number of items to be completed varied by user group: combustible cigarette smokers (10 items), other tobacco product users (9 items), and non-smokers (8 items for combustible cigarette assessments or 7 items for other product assessments).

Prior to the main survey, a pilot test of the Attractiveness Scale was conducted in a sample of 113 participants (21 non-smokers and 92 smokers) to assess item clarity and preliminary internal consistency. The Cronbach’s *α* in the pilot sample was 0.878, indicating good preliminary reliability. In the total sample, the overall Cronbach’s α for the scale was 0.701.

### 2.5 Data collection

Data were collected between June 19 and August 30, 2025. Eligible participants were first screened according to the inclusion criteria. During questionnaire completion, time limits were applied to each item to ensure adequate reading and reflection. Attention-check questions were embedded throughout the survey to identify and exclude inattentive or low-quality responses. Additionally, dedicated staff conducted logical and quality checks on all submitted responses to ensure data quality and overall reliability.

### 2.6 Statistical analysis

Statistical analysis was performed using R software. The overall demographic characteristics, cognitive ratings, and attractiveness ratings were described and compared between non-smokers and smokers. Descriptive statistics (mean ± standard deviation, SD) for appeal ratings of five tobacco products were also stratified by non-smokers and smokers. A linear mixed-model examined influencing factors, incorporating 13 independent variables including region, gender, age, tobacco product history, and risk perception ratings.

### 2.7 Ethical considerations

This study was approved by the Ethics Committee of the Shanghai Institute for Biomedical and Pharmaceutical Technologies. All participants first read the electronic version of the informed consent form on the online survey platform. Those who consented to participate clicked “I have read and agree to participate” to proceed to the questionnaire interface.

## 3 Results

### 3.1 Basic information

A total of 15,601 participants (9,700 non-smokers; 5,901 smokers). Age distribution differed markedly: non-smokers were more concentrated in 18–20 years (20.08% vs. 5.86%), whereas smokers were more common in 31–40 years (39.28% vs. 25.47%) and ≥41 years (10.47% vs. 8.80%) (*p* < 0.001). Education also varied (*p* < 0.001): smokers had a higher share with college degrees (20.35% vs. 17.13%) but fewer with ≥Master’s degrees (6.02% vs. 9.63%). Occupation differed significantly (*p* < 0.001), with more smokers among non-government employees and self-employed individuals, while non-smokers included many more college students. Income distributions also differed (*p* < 0.001) (Table 1).

**Table 1:** Demographic characteristics (*n* = 15,601).

Variables	Total (*n* = 15,601)	Non-smoker (*n* = 9,700)	Smokers (*n* = 5,901)	*p*
Age				<0.001
18–20	2,294 (14.70)	1948 (20.08)	346 (5.86)	
21–30	7,046 (45.16)	4,427 (45.64)	2,619 (44.38)	
31–40	4,789 (30.70)	2,471 (25.47)	2,318 (39.28)	
≥41	1,472 (9.44)	854 (8.80)	618 (10.47)	
Education				<0.001
≤Junior high school	236 (1.51)	142 (1.46)	94 (1.59)	
High /vocational school	1,203 (7.71)	760 (7.84)	443 (7.51)	
College degree	2,863 (18.35)	1,662 (17.13)	1,201 (20.35)	
Undergraduate degree	10,010 (64.16)	6,202 (63.94)	3,808 (64.53)	
≥Master’s degree	1,289 (8.26)	934 (9.63)	355 (6.02)	
Occupation				<0.001
Government employees	1,268 (8.13)	594 (6.12)	674 (11.42)	
Non-government employees	7,357 (47.16)	4,126 (42.54)	3,231 (54.75)	
Self-employed individuals	2,329 (14.93)	1,187 (12.24)	1,142 (19.35)	
College students	3,541 (22.70)	2,980 (30.72)	561 (9.51)	
Others	1,106 (7.09)	813 (8.38)	293 (4.97)	
Monthly household income				<0.001
<2000	1,594 (10.22)	1,265 (13.04)	329 (5.58)	
2001–3,000	2009 (12.88)	1,412 (14.56)	597 (10.12)	
3,001–5,000	3,728 (23.90)	2,323 (23.95)	1,405 (23.81)	
5,001–8,000	4,209 (26.98)	2,342 (24.14)	1867 (31.64)	
≥8,000	4,061 (26.03)	2,358 (24.31)	1703 (28.86)	

Table 2 description and compared perceptions (mean ± SD) across five products, using conventional cigarettes (CC) as the reference. CC had the highest familiarity (3.97 ± 1.04). All alternative products scored significantly lower on familiarity. CC was also rated highest for perceived health risk (4.38) and addictiveness (4.22). Withdrawal severity and perceived social support were consistently lower for all alternatives than for CC (all *p* < 0.05 vs. CC). In addition, across all five products, smokers reported significantly higher familiarity than non-smokers (e.g., CC 4.45 vs. 3.68; EC 3.56 vs. 3.06), and consistently higher perceived social support (e.g., CC 3.83 vs. 3.11). In contrast, smokers generally perceived lower health risk for every product (e.g., CC 4.14 vs. 4.52; TFONP 2.68 vs. 3.16), as well as lower addictiveness and lower withdrawal severity compared with non-smokers. These between-group differences were statistically significant (*p* < 0.05) across most metrics and products, indicating a systematic tendency for smokers to view all products as more familiar and socially supported, but less risky and less dependence-forming than non-smokers (Supplementary Table S2).

**Table 2:** Perception ratings of five tobacco products (Mean ± SD).

Variables	CC	EC	HTP	ONP	TFONP
Familiarity	3.97 ± 1.04	3.25 ± 1.06*	2.18 ± 1.01*	2.01 ± 1.07*	1.81 ± 0.95*
Perceived health risk	4.38 ± 0.76	3.57 ± 1.05*	4.1 ± 0.81*	3.73 ± 0.99*	2.98 ± 1.2*
Perceived addictiveness	4.22 ± 0.87	3.31 ± 1.01*	3.29 ± 1.02*	3.25 ± 1.06*	2.94 ± 1.12*
Perceived severity of withdrawal	3.05 ± 1.03	2.28 ± 1.09*	2.63 ± 1.01*	2.56 ± 1.05*	2.23 ± 1.17*
Perceived social support	3.38 ± 1.17	2.5 ± 1.05*	2.51 ± 1.03*	2.39 ± 1.06*	2.48 ± 1.08*

All significance tests used CC as the control group. * Indicates *p* < 0.05.

### 3.2 Attractiveness ratings

Table 3 reported attractiveness across decision-making, sociocultural, engineering, and sensory domains. Overall total attractiveness scores were similar for CC and EC (both 3.04), while HTP, ONP, and TFONP had significantly lower totals (2.59–2.63; *p* < 0.05 vs. CC). By domain, EC scored higher than CC for “feel & sensation” (3.56 vs. 2.84) and “design” (3.33 vs. 2.98), and slightly higher for convenience (3.46 vs. 3.31), indicating stronger product/experience appeal. In contrast, sociocultural “social tool” ratings were lower for EC and other alternatives than for CC. For physiological satisfaction, CC remained highest (3.78), with EC/other products lower.

**Table 3:** Attractiveness ratings of five tobacco products (Mean ± SD).

Variables	CC	EC	HTP	ONP	TFONP
Health risk decision-making: Self-health	2.79 ± 1.39	2.9 ± 1.32*	2.53 ± 1.28*	2.52 ± 1.34*	2.76 ± 1.40*
Health risk decision-making: Other-health	2.77 ± 1.39	2.9 ± 1.30*	2.57 ± 1.27*	2.67 ± 1.33*	2.83 ± 1.36*
Sociocultural factors: Social tool	3.29 ± 1.34	2.38 ± 1.19*	2.32 ± 1.14*	2.20 ± 1.15*	2.27 ± 1.15*
Sociocultural factors: Cultural symbol	2.63 ± 1.22	2.88 ± 1.31*	2.5 ± 1.19*	2.29 ± 1.18*	2.31 ± 1.15*
Engineering attributes: Convenience	3.31 ± 1.23	3.46 ± 1.23*	2.68 ± 1.16*	2.79 ± 1.26*	2.80 ± 1.24*
Engineering attributes: Use method	3.09 ± 1.34	2.76 ± 1.19*	2.86 ± 1.24*	3.12 ± 1.33*	3.04 ± 1.32*
Engineering attributes: Design	2.98 ± 1.12	3.33 ± 1.23*	2.76 ± 1.16*	2.50 ± 1.12*	2.45 ± 1.11*
Engineering attributes: specification	3.2 ± 1.29	–	–	–	–
Neurosensory experience: Feel & Sensation	2.84 ± 1.41	3.56 ± 1.31*	2.75 ± 1.22*	2.60 ± 1.20*	2.55 ± 1.19*
Neurosensory experience: Physiological satisfaction	3.78 ± 1.19	3.40 ± 1.09*	3.50 ± 1.12*	3.35 ± 1.14*	3.34 ± 1.15*
Total score	3.04 ± 1.33	3.04 ± 1.31	2.63 ± 1.22*	2.59 ± 1.27*	2.63 ± 1.27*

All significance tests used CC as the control group. * Indicates *p* < 0.05.

**Figure 1:**
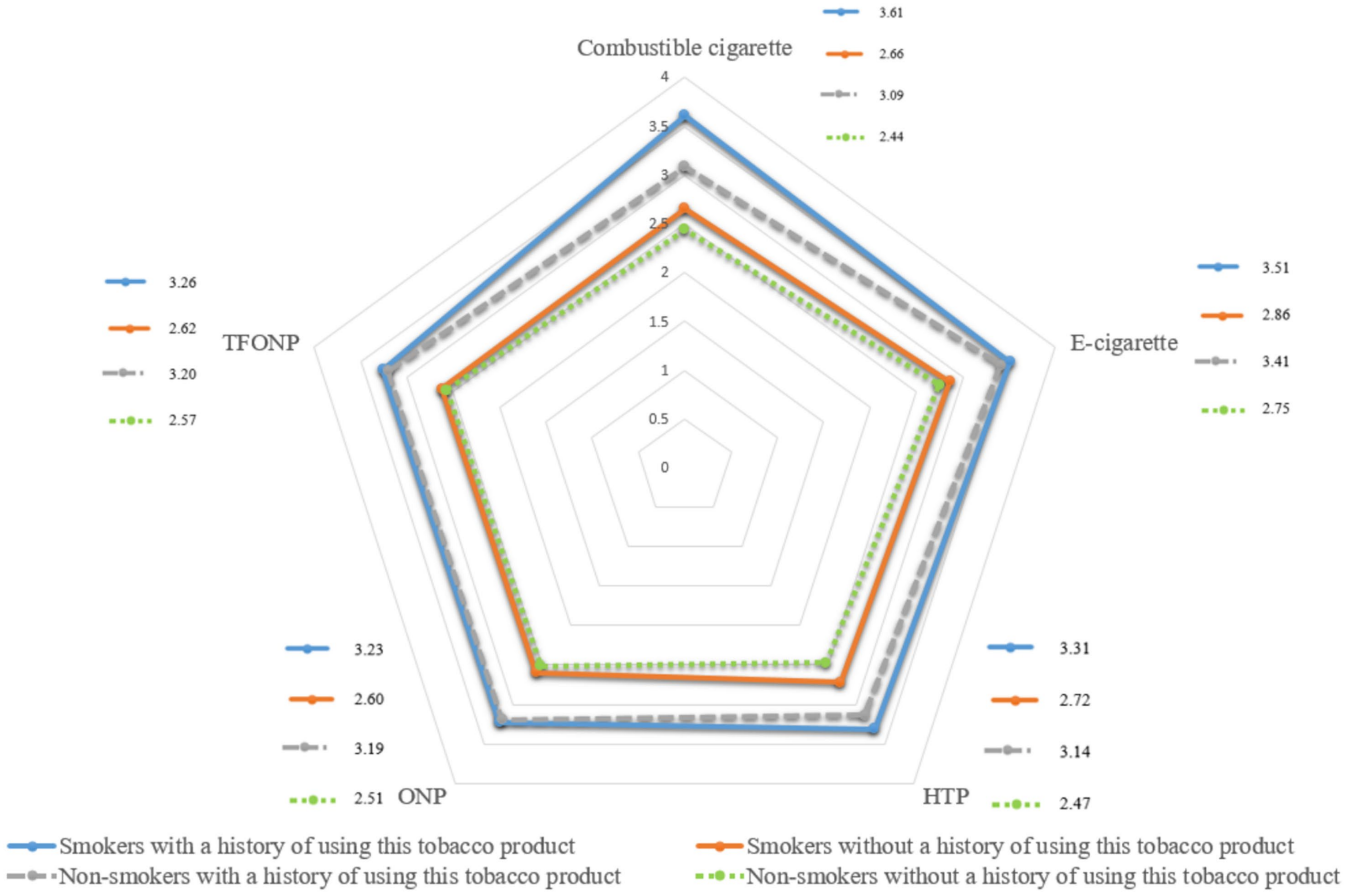
Attractiveness ratings of five tobacco products among smokers and non-smokers.

Attractiveness scores were consistently higher among smokers than non-smokers, especially for conventional cigarettes (CC) and e-cigarettes (EC). Among participants with use history, smokers scored higher on multiple domains (health-risk decision-making, sociocultural appeal, engineering features, and physiological satisfaction), yielding higher total ratings (e.g., CC 3.61 vs. 3.09; EC 3.51 vs. 3.41). Differences for ONP/TFONP were smaller and sometimes non-significant, suggesting weaker smoker–non-smoker separation for newer oral products. Among participants without use history, absolute scores were lower overall, yet smokers still tended to rate products as more attractive, including higher total scores across product categories (e.g., CC 2.66 vs. 2.44; HTP 2.71 vs. 2.47) (Figure 1; Supplementary Table S3).

### 3.3 Factors analysis of attractiveness

Across the total sample, Model 1 (without perceived health risks) showed no statistically significant difference in attractiveness ratings between smokers and non-smokers (*β* = 0.001, *p* = 0.896). In contrast, Model 2 (with perceived health risks) revealed a small but significant negative association between smoking status and attractiveness (β = −0.06, *p* < 0.001), indicating that at the same level of perceived health risk, smokers received lower attractiveness ratings than non-smokers. In Model 2, perceived health risk itself was also negatively associated with attractiveness (*β* = −0.26, *p* < 0.001). In addition, most other covariates were highly consistent across models: product type effects were stable (EC higher; HTP/NOP/TFONP lower vs. CC), and history of use remained the strongest positive predictor (*β* = 0.56 in both, *p* < 0.001). Perception variables were robustly associated with attractiveness in Model 1 (e.g., familiarity, addictiveness, social support positive; health risks negative) (Table 4).

**Table 4:** Analysis of influencing factors of the attractiveness (total sample).

Variables	Model 1			Model 2		
	*β*	SE	*P*	*β*	SE	*P*
Smoker						
No	Control			Control		
Yes	0.001	0.01	0.896	−0.06	0.01	<0.001
Tobacco product						
CC	Control			Control		
EC	0.14	0.01	<0.001	0.14	0.01	<0.001
HTP	−0.12	0.01	<0.001	−0.12	0.01	<0.001
NOP	−0.14	0.01	<0.001	−0.14	0.01	<0.001
TFONP	−0.09	0.01	<0.001	−0.10	0.01	<0.001
History of use						
No	Control			Control		
Yes	0.56	0.01	<0.001	0.56	0.01	<0.001
Area						
Beijing	Control			Control		
Shanghai	0.02	0.02	0.222	0.02	0.02	0.140
Guangzhou	0.02	0.02	0.216	0.01	0.02	0.393
Shenzhen	0.04	0.02	0.010	0.04	0.02	0.012
Chengdu	0.06	0.02	<0.001	0.05	0.02	0.001
Zhengzhou	0.07	0.02	<0.001	0.06	0.02	<0.001
Gender						
Female	Control			Control		
Male	−0.03	0.01	0.005	−0.03	0.01	0.002
Age group						
18–20	Control			Control		
21–30	0.04	0.02	0.013	0.04	0.02	0.013
31–40	0.08	0.02	<0.001	0.07	0.02	0.001
≥41	0.05	0.02	0.044	0.03	0.02	0.275
Education						
≤Junior high school	Control			Control		
High/vocational	−0.05	0.04	0.209	−0.05	0.04	0.182
College degree	0.01	0.04	0.735	0.01	0.04	0.767
Undergraduate	0.001	0.04	0.924	−0.01	0.04	0.820
≥Master’s degree	−0.11	0.04	0.014	−0.10	0.04	0.014
Occupation						
Government employees	Control			Control		
Non-governmental employees	0.07	0.02	<0.001	0.06	0.02	0.001
Self-employed	0.07	0.02	0.001	0.06	0.02	0.003
College student	0.001	0.02	0.880	0.01	0.02	0.654
Others	0.05	0.02	0.037	0.05	0.02	0.029
Monthly income						
<2000	Control			Control		
2001–3,000	0.02	0.02	0.314	0.02	0.02	0.336
3,001–5,000	0.03	0.02	0.149	0.02	0.02	0.183
5,001–8,000	0.002	0.02	0.902	−0.002	0.02	0.923
≥8,000	−0.02	0.02	0.298	−0.02	0.02	0.311
Familiarity	0.19	0.01	<0.001	0.18	0.01	<0.001
Perceived addictiveness	0.05	0.01	<0.001	0.10	0.01	<0.001
Perceived severity of withdrawal	−0.11	0.01	<0.001	−0.07	0.01	<0.001
Perceived social support	0.25	0.01	<0.001	0.22	0.01	<0.001
Perceived health risks	–	–	–	−0.26	0.01	<0.001

Stratified analysis by current smoking status showed that use history was the strongest positive correlate of attractiveness in both groups (*β* = 0.35 in non-smokers; β = 0.53 in smokers; both *p* < 0.001). Product type effects differed by smoking status: among non-smokers, EC was rated more attractive than CC (β = 0.25, *p* < 0.001), while HTP and NOP were less attractive; TFONP was slightly higher. Among smokers, all non-cigarette products (EC/HTP/NOP/TFONP) had lower attractiveness than CC (all *p* < 0.001). Across both groups, greater familiarity, perceived addictiveness and social support were positively associated with attractiveness, whereas perceived health risks and withdrawal severity were negatively associated, whereas perceived health risks were negatively associated (stronger in non-smokers: *β* = −0.37 vs. −0.06) (Supplementary Table S4). Overall, the stratified analyses were broadly consistent with the overall-population model regarding the direction and statistical significance of key predictors; however, the age-group effect differed across strata, and effect sizes varied slightly by smoking status.

## 4 Discussion

This study examined perceived attractiveness of five tobacco/nicotine product types (combustible cigarettes, e-cigarettes, heated tobacco products, oral nicotine products, and tobacco-free oral nicotine pouches) among a large sample of smartphone-using adults. Our findings show clear product- and user-group differences in perceived appeal: e-cigarettes were rated as most appealing among non-smokers and among smokers without prior e-cigarette experience, whereas combustible cigarettes remained most appealing among combustible-cigarette smokers. These patterns are broadly consistent with prior research showing that e-cigarettes may be perceived as appealing due to features such as flavors, accessibility/convenience, and the social media environment (32, 33). While e-cigarette appeal has been widely discussed in the literature, our contribution lies in providing a within-respondent, multi-product comparison using a designed attractiveness scale across five product categories. In particular, we extend the comparative evidence base by including oral nicotine products and tobacco-free oral nicotine pouches alongside cigarettes, e-cigarettes, and HTPs, and by examining appeal patterns and correlates across smoking/use-history subgroups. This multi-product perspective helps clarify where novel products may rank in perceived attractiveness relative to cigarettes and e-cigarettes, which is directly relevant to anticipating substitution versus uptake among nicotine-naïve individuals.

The prior use of tobacco/nicotine products was strongly associated with higher attractiveness ratings in our study, and e-cigarette use—particularly current use—was positively associated with susceptibility to tobacco product use (34). We also observed that higher perceived health risk was associated with lower attractiveness, whereas greater familiarity with tobacco products, perceived addictiveness, perceived severity of withdrawal, and perceived social support were associated with higher attractiveness. These findings suggest that attractiveness is not driven solely by sensory or marketing cues, but also reflects cognitive appraisals (e.g., risk and dependence perceptions) and social-context factors that may shape perceived desirability and intentions. Prior studies have similarly shown that communication environments can shift perceived appeal and risk—for example, pictorial warnings reducing attractiveness and increasing perceived risks, and modified-risk claims reducing perceived risk without necessarily increasing appeal (14, 19). However, given the cross-sectional design, these associations should not be interpreted causally (i.e., use history causing higher attractiveness). A more plausible interpretation is that higher perceived attractiveness increases the likelihood of trial and continued use; following experimentation, direct experience with the product (e.g., sensory satisfaction or perceived benefits) may further reinforce perceived appeal. This potential feedback process is consistent with prior literature linking product appeal, perceived benefits/risk, and marketing receptivity to susceptibility and use behaviors (19, 35). Longitudinal or experimental research is needed to determine temporal ordering and causal mechanisms.

Furthermore, our analysis indicated that perceived health risks influenced the observed association between smoking status and attractiveness. To better characterize the difference between smokers and non-smokers, we fitted two models. In Model 1, which adjusted for sociodemographic and product-related covariates but not for perceived health risks, smoking status showed no meaningful association with attractiveness. In contrast, Model 2 additionally adjusted for perceived health risks, which was strongly and inversely associated with attractiveness; after this adjustment, smoking status became modestly but statistically significant. The divergence between the two models indicated that perceived health risk was an important correlate of both smoking status and product appeal (36). This pattern suggested that risk perceptions were closely intertwined with the smoking–appeal association and meaningfully altered the estimated effect of smoking, consistent with evidence that perceived risks are closely linked to product appeal and consumer responses (19, 22). Our univariate analysis revealed that Smokers scored low on health risk perception but high on attractiveness, and stratified analyses further revealed a stronger negative association between risk perception and attractiveness among non-smokers than among smokers. Future longitudinal research should examine how risk perceptions and appeal co-vary over time and evaluate their relative contributions to smoking-related differences in perceived attractiveness (37).

Because attractiveness is closely tied to experimentation and potential uptake, our findings have several practical implications for public communication and health education. First, the consistently high appeal of e-cigarettes among non-smokers highlights the importance of providing clear and comprehensive information about product characteristics, including novelty, perceived social benefits, and risk-related attributes, particularly for youth and other nicotine-naïve groups (33, 35, 38). Second, the relatively high appeal of tobacco-free oral nicotine pouches among non-smokers without prior use history suggests that descriptors such as “tobacco-free” may shape perceptions of product acceptability and risk, underscoring the need for transparent communication that enables consumers to better understand what such descriptors do and do not imply (34, 39). Communication materials should therefore present balanced, evidence-based information on nicotine dependence and potential health risks, allowing individuals to interpret these attributes in an informed manner. Third, given the observed association between perceived risk and attractiveness, providing accessible and accurate information on health risks may support consumers in making more informed and reasoned decisions regarding the use of different tobacco and nicotine products (40, 41).

A key strength of this study is its within-respondent, multi-product design, which allowed for standardized comparisons of five tobacco/nicotine products in a large sample of smartphone-using adults. However, several limitations should be noted. First, this was an online, non-probability survey that skewed toward younger and more highly educated respondents with greater internet access (42) so findings should not be interpreted as nationally representative prevalence estimates and may not generalize to adults with limited internet access. Second, the cross-sectional design precludes causal inference, and reverse causation is possible (e.g., higher perceived attractiveness may precede trial and continued use). Third, measures were self-reported and may be subject to recall and social-desirability biases. Future studies using probability-based sampling, longitudinal follow-up, and experimental designs would strengthen external validity and causal interpretation.

## 5 Conclusion

This multicenter study of a large sample of smartphone-using adults in China revealed distinct patterns in perceived appeal across five tobacco/nicotine product types. Among smokers with a history of CC use, combustible cigarettes remained the most appealing, whereas e-cigarettes received the highest appeal ratings among non-smokers. Prior product use history showed the strongest association with attractiveness. Notably, adjustment for perceived health risks changed the association between smoking status and attractiveness ratings, strengthening the observed negative relationship. Perceived health risks were inversely associated with appeal, while greater familiarity and higher perceived social support were positively associated with appeal.

## Data Availability

The raw data supporting the conclusions of this article will be made available by the authors, without undue reservation.
