# Supplementary material for: Attractiveness and determinants of different tobacco products among Chinese smokers and non-smokers: a web-based cross-sectional study
Source: Front Public Health. 2026 Feb 24;14:1763854. doi: 10.3389/fpubh.2026.1763854 (PMC12971477; doi:10.3389/fpubh.2026.1763854)
Supplement: Supplementary file 1 [file Table_1.docx]

Table S1A Items of attractiveness survey

| Dimension | Item |
| --- | --- |
| Health risk decision-making | 1. Self-health: Impact on one's own health |
|  | 2. Other-health: Impact on others' health |
| Sociocultural factors | (1) Social tool: Used as a social tool in my daily work or social engagements. |
|  | (2) Cultural symbol: Using this tobacco product looks cool. |
|  | (1) Convenience: Ease of carrying and use |
| Product engineering attributes | (2) Use method:  CCs involve combustion via open flame, producing smoke;  E-cigarettes utilize heating devices to vaporize e-liquid, generating smoke-like vapor;  HTPs use smart devices to heat tobacco without combustion, yet produce smoke-like vapor;  ONP or TFONP in the mouth offers a more discreet. |
|  | (3) Product Design: Visual Design |
|  | (4) Specification selection: CCs offer slim and regular options (Note: This item does not apply to the other four tobacco products). |
| Neurosensory experience | (1) Feel & Sensation |
|  | (2) Physiological satisfaction |

Table S1B Question: Based on your perspective or experience, rate the appeal of combustible cigarette (1 = “None” to 5 = “Extremely high”) (Please fill in the corresponding table field)

|  | Extremely high appealing (5) | More appealing (4) | Generally  (3) | Not very appealing (2) | No appealing  (1) |
| --- | --- | --- | --- | --- | --- |
| a. The Impact of combustible cigarette on personal health |  |  |  |  |  |
| b. In my daily work or social settings, combustible cigarette can serve as a social tool |  |  |  |  |  |
| c. Combustible cigarettes are easy to carry and use |  |  |  |  |  |
| …… |  |  |  |  |  |
| …… |  |  |  |  |  |
| …… |  |  |  |  |  |

Table S2 Perception ratings of five tobacco products among smokers and non-smokers (Mean ± SD)

|  | CC | | EC | | HTP | | ONP | | TFONP | |
| --- | --- | --- | --- | --- | --- | --- | --- | --- | --- | --- |
|  | Non-smokers | Smokers | Non-smokers | Smokers | Non-smokers | Smokers | Non-smokers | Smokers | Non-smokers | Smokers |
| Familiarity | 3.68±1.07 | 4.45±0.80* | 3.06±1.05 | 3.56±1.01* | 1.98±0.94 | 2.50±1.04* | 1.82±0.99 | 2.31±1.13* | 1.63±0.84 | 2.09±1.05* |
| Perceived health risk | 4.52±0.70 | 4.14±0.78* | 3.70±1.01 | 3.37±1.07* | 4.28±0.75 | 3.82±0.82* | 3.90±0.96 | 3.44±0.98* | 3.16±1.21 | 2.68±1.13* |
| Perceived addictiveness | 4.24±0.87 | 4.20±0.87* | 3.44±0.99 | 3.11±1.00* | 3.40±1.02 | 3.12±0.99* | 3.38±1.05 | 3.03±1.05* | 3.05±1.12 | 2.75±1.08* |
| Perceived severity of withdrawal | 3.13±0.98 | 2.90±1.09* | 2.46±1.04 | 1.98±1.11* | 2.78±0.97 | 2.39±1.03* | 2.72±1.01 | 2.29±1.07* | 2.39±1.15 | 1.97±1.16* |
| Perceived social support | 3.11±1.19 | 3.83±0.99* | 2.42±1.02 | 2.63±1.09* | 2.43±1.01 | 2.66±1.04* | 2.31±1.03 | 2.52±1.09* | 2.43±1.07 | 2.57±1.09* |

Note: All significance tests used non-smokers as the control group. * indicates P < 0.05, indicating a significant difference.

Table S3 Attractiveness ratings of five tobacco products among smokers and non-smokers (Mean ± SD)

|  | CC | | EC | | HTP | | ONP | | TFONP | |
| --- | --- | --- | --- | --- | --- | --- | --- | --- | --- | --- |
|  | Non-smokers | Smokers | Non-smokers | Smokers | Non-smokers | Smokers | Non-smokers | Smokers | Non-smokers | Smokers |
| **Participants with use history** |  |  |  |  |  |  |  |  |  |  |
| Health risk decision-making: Self-health | 2.81±1.35 | 3.18±1.19* | 3.25±1.19 | 3.35±1.10* | 2.94±1.23 | 3.15±1.11* | 3.09±1.16 | 3.19±1.20 | 3.24±1.23 | 3.33±1.26 |
| Health risk decision-making: Other-health | 2.81±1.40 | 2.98±1.20* | 3.09±1.20 | 3.21±1.16* | 2.87±1.20 | 3.11±1.12* | 3.22±1.21 | 3.22±1.20 | 3.24±1.15 | 3.38±1.16 |
| Sociocultural factors: Social tool | 3.45±1.24 | 3.99±1.02* | 2.64±1.17 | 2.75±1.24* | 3.07±1.24 | 3.04±1.20 | 2.92±1.28 | 2.93±1.21 | 2.94±1.16 | 2.99±1.19 |
| Sociocultural factors: Cultural symbol | 2.76±1.13 | 3.23±1.07* | 3.49±1.21 | 3.45±1.20 | 3.03±1.19 | 3.23±1.14* | 3.09±1.22 | 3.08±1.20 | 2.99±1.27 | 3.04±1.21 |
| Engineering attributes: Convenience | 3.43±1.11 | 3.91±0.97* | 3.95±1.02 | 4.02±0.98* | 3.21±1.17 | 3.29±1.09 | 3.38±1.14 | 3.30±1.19 | 3.36±1.22 | 3.38±1.15 |
| Engineering attributes: Use method | 3.20±1.26 | 3.72±1.07* | 3.13±1.10 | 3.36±1.06* | 3.25±1.16 | 3.51±1.08* | 3.56±1.14 | 3.62±1.17 | 3.56±1.20 | 3.56±1.11 |
| Engineering attributes: Design | 3.08±1.02 | 3.46±1.00* | 3.81±1.05 | 3.83±1.02 | 3.26±1.08 | 3.39±1.07 | 2.99±1.15 | 3.10±1.14 | 3.06±1.08 | 3.08±1.13 |
| Engineering attributes: Specification | 3.36±1.20 | 3.83±1.04* | - | - | - | - | - | - | - | - |
| Neurosensory experience | 2.81±1.36 | 3.69±1.09* | 4.14±1.03 | 4.14±1.01 | 3.33±1.06 | 3.50±1.09* | 3.22±1.12 | 3.27±1.10 | 3.13±1.11 | 3.19±1.15 |
| Neurosensory experience: Physiological satisfaction | 3.18±1.30 | 4.12±0.97* | 3.24±1.12 | 3.45±1.08* | 3.34±1.20 | 3.54±1.10* | 3.21±1.15 | 3.38±1.14* | 3.27±1.14 | 3.36±1.16 |
| **Total score** | **3.09±0.74** | **3.61±0.57*** | **3.41±0.64** | **3.51±0.60*** | **3.14±0.72** | **3.31±0.6*8** | **3.19±0.73** | **3.23±0.71** | **3.20±0.70** | **3.26±0.72** |
| **Participants without use history** |  |  |  |  |  |  |  |  |  |  |
| Health risk decision-making: Self-health | 2.43±1.47 | 2.71±1.26 | 2.65±1.40 | 2.75±1.18* | 2.41±1.36 | 2.56±1.10* | 2.44±1.41 | 2.52±1.21* | 2.71±1.45 | 2.74±1.30 |
| Health risk decision-making: Other-health | 2.54±1.51 | 2.84±1.27* | 2.74±1.37 | 2.80±1.21* | 2.47±1.33 | 2.57±1.14* | 2.63±1.38 | 2.62±1.23 | 2.80±1.40 | 2.79±1.28 |
| Sociocultural factors: Social tool | 2.59±1.29 | 2.92±1.30* | 2.20±1.13 | 2.20±1.12 | 2.18±1.09 | 2.33±1.11* | 2.11±1.11 | 2.22±1.15* | 2.21±1.12 | 2.26±1.14* |
| Sociocultural factors: Cultural symbol | 2.03±1.10 | 2.42±1.09* | 2.57±1.26 | 2.63±1.24 | 2.32±1.15 | 2.61±1.16* | 2.18±1.15 | 2.34±1.14* | 2.22±1.13 | 2.33±1.12* |
| Engineering attributes: Convenience | 2.71±1.20 | 2.72±1.13 | 3.14±1.26 | 3.32±1.18* | 2.52±1.14 | 2.77±1.14* | 2.70±1.26 | 2.84±1.24* | 2.72±1.25 | 2.83±1.21* |
| Engineering attributes: Use method | 2.46±1.31 | 2.66±1.20 | 2.43±1.14 | 2.69±1.11* | 2.70±1.26 | 2.96±1.18* | 3.06±1.35 | 3.10±1.31 | 3.01±1.33 | 3.01±1.29 |
| Engineering attributes: Design | 2.51±1.08 | 2.36±1.23 | 3.06±1.26 | 3.16±1.22* | 2.62±1.15 | 2.83±1.12* | 2.42±1.11 | 2.51±1.09* | 2.37±1.10 | 2.47±1.09* |
| Engineering attributes: Specification | 2.57±1.23 | 2.79±1.25* | - | - | - | - | - | - | - | - |
| Neurosensory experience | 2.09±1.26 | 2.50±1.32 | 3.24±1.36 | 3.33±1.30* | 2.50±1.20 | 3.00±1.16* | 2.52±1.19 | 2.60±1.19* | 2.50±1.19 | 2.53±1.17 |
| **Total score** | **2.44±0.84** | **2.66±0.73*** | **2.75±0.86** | **2.86±0.77*** | **2.47±0.81** | **2.71±0.73*** | **2.51±0.83** | **2.60±0.78*** | **2.57±0.86** | **2.62±0.80*** |

Note: All significance tests used non-smokers as the control group. * indicates P < 0.05, indicating a significant difference.

Table S4 Analysis of influencing factors of the attractiveness ratings among smokers and non-smokers

|  | Non-smokers | | | | Smokers | | | |
| --- | --- | --- | --- | --- | --- | --- | --- | --- |
|  | β | SE | t | P | β | SE | t | P |
| Area |  |  |  |  |  |  |  |  |
| Beijing | control |  |  |  | control |  |  |  |
| Shanghai | 0.01 | 0.02 | 0.64 | 0.523 | 0.05 | 0.02 | 2.17 | 0.030 |
| Guangzhou | 0.02 | 0.02 | 0.73 | 0.468 | 0.01 | 0.02 | 0.35 | 0.728 |
| Shenzhen | 0.05 | 0.02 | 2.57 | 0.010 | 0.02 | 0.02 | 0.73 | 0.462 |
| Chengdu | 0.05 | 0.02 | 2.37 | 0.018 | 0.06 | 0.02 | 2.63 | 0.009 |
| Zhengzhou | 0.08 | 0.02 | 3.59 | < 0.001 | 0.04 | 0.02 | 1.76 | 0.078 |
| Gender |  |  |  |  |  |  |  |  |
| Female | control |  |  |  | control |  |  |  |
| Male | -0.03 | 0.01 | -2.35 | 0.019 | -0.02 | 0.01 | -1.57 | 0.116 |
| Tobacco product |  |  |  |  |  |  |  |  |
| CC | control |  |  |  | control |  |  |  |
| EC | 0.25 | 0.01 | 37.19 | < 0.001 | -0.13 | 0.01 | -12.86 | < 0.001 |
| HTP | -0.06 | 0.01 | -8.22 | < 0.001 | -0.35 | 0.01 | -28.02 | < 0.001 |
| NOP | -0.02 | 0.01 | -3.41 | 0.001 | -0.46 | 0.01 | -35.33 | < 0.001 |
| TFONP | 0.04 | 0.01 | 5.08 | < 0.001 | -0.44 | 0.01 | -33.20 | < 0.001 |
| History of use |  |  |  |  |  |  |  |  |
| No | control |  |  |  | control |  |  |  |
| Yes | 0.35 | 0.01 | 39.03 | < 0.001 | 0.53 | 0.01 | 50.84 | < 0.001 |
| Age group |  |  |  |  |  |  |  |  |
| 18-20 | control |  |  |  | control |  |  |  |
| 21-30 | 0.06 | 0.02 | 2.75 | 0.006 | -0.05 | 0.03 | -1.67 | 0.095 |
| 31-40 | 0.08 | 0.03 | 3.19 | 0.001 | -0.02 | 0.03 | -0.75 | 0.454 |
| ≥41 | 0.01 | 0.03 | 0.47 | 0.635 | -0.03 | 0.04 | -0.93 | 0.352 |
| Education |  |  |  |  |  |  |  |  |
| ≤Junior high school | control |  |  |  | control |  |  |  |
| High/vocational school | -0.04 | 0.06 | -0.64 | 0.520 | -0.05 | 0.05 | -0.94 | 0.348 |
| College degree | 0.04 | 0.05 | 0.77 | 0.443 | -0.01 | 0.05 | -0.24 | 0.812 |
| Undergraduate | -0.01 | 0.05 | -0.12 | 0.903 | 0.02 | 0.05 | 0.50 | 0.619 |
| ≥Master's degree | -0.08 | 0.06 | -1.46 | 0.143 | -0.07 | 0.06 | -1.17 | 0.241 |
| Occupation |  |  |  |  |  |  |  |  |
| Government employees | control |  |  |  | control |  |  |  |
| Non-governmental employees | 0.06 | 0.03 | 2.44 | 0.015 | 0.05 | 0.02 | 2.52 | 0.012 |
| Self-employed | 0.10 | 0.03 | 3.37 | 0.001 | 0.01 | 0.02 | 0.31 | 0.756 |
| College student | 0.02 | 0.03 | 0.57 | 0.569 | 0.02 | 0.03 | 0.72 | 0.473 |
| Others | 0.08 | 0.03 | 2.34 | 0.019 | -0.03 | 0.03 | -0.80 | 0.424 |
| Monthly income | | | | |  |  |  |  |
| <2000 | control |  |  |  | control |  |  |  |
| 2001-3000 | 0.01 | 0.02 | 0.38 | 0.704 | 0.04 | 0.03 | 1.39 | 0.166 |
| 3001-5000 | 0.00 | 0.02 | 0.04 | 0.968 | 0.09 | 0.03 | 3.13 | 0.002 |
| 5001-8000 | -0.01 | 0.02 | -0.56 | 0.575 | 0.05 | 0.03 | 1.75 | 0.081 |
| ≥8000 | -0.02 | 0.02 | -0.97 | 0.334 | 0.03 | 0.03 | 0.85 | 0.396 |
| Familiarity | 0.21 | 0.01 | 22.50 | < 0.001 | 0.13 | 0.01 | 13.37 | < 0.001 |
| Perceived addictiveness | 0.09 | 0.01 | 8.88 | < 0.001 | 0.15 | 0.01 | 13.43 | < 0.001 |
| Perceived severity of withdrawal | -0.07 | 0.01 | 6.97 | < 0.001 | -0.06 | 0.01 | 6.58 | < 0.001 |
| Perceived social support | 0.21 | 0.01 | 24.14 | < 0.001 | 0.19 | 0.01 | 18.81 | < 0.001 |
| Perceived health risks | -0.37 | 0.01 | -35.44 | < 0.001 | -0.06 | 0.01 | -5.27 | < 0.001 |
